# The TβRI promotes migration and metastasis through thrombospondin 1 and ITGAV in prostate cancer cells

**DOI:** 10.1038/s41388-024-03165-3

**Published:** 2024-09-20

**Authors:** Yabing Mu, Anders Wallenius, Guangxiang Zang, Shaochun Zhu, Stina Rudolfsson, Karthik Aripaka, Anders Bergh, André Mateus, Maréne Landström

**Affiliations:** 1 Department of Medical Bioscience, Umeå University, Umeå, Sweden; 2 Department of Chemistry, Umeå University, Umeå, Sweden; 3 Molecular Infection Medicine Sweden, Umeå University, Umeå, Sweden

**Keywords:** Tumour biomarkers, Predictive markers

## Abstract

TGFβ potently modifies the extracellular matrix (ECM), which is thought to favor tumor cell invasion. However, the mechanism whereby the cancer cells employ the ECM proteins to facilitate their motility is largely unknown. In this study we used RNA-seq and proteomic analysis to examine the proteins secreted by castration-resistant prostate cancer (CRPC) cells upon TGFβ treatment and found that thrombospondin 1 (THBS1) was observed to be one of the predominant proteins. The CRISPR Cas9, or siRNA techniques was used to downregulate TGFβ type I receptor (TβRI) to interfere with TGFβ signaling in various cancer cells in vitro. The interaction of ECM proteins with the TβRI in the migratory prostate cancer cells in response to TGFβ1 was demonstrated by several different techniques to reveal that THBS1 mediates cell migration by interacting with integrin subunit alpha V (ITGAV) and TβRI. Deletion of TβRI or THBS1 in cancer cells prevented their migration and invasion. THBS1 belongs to a group of tumorigenic ECM proteins induced via TGFβ signaling in CRPC cells, and high expression of THBS1 in human prostate cancer tissues correlated with the degree of malignancy. TGFβ-induced production of THBS1 through TβRI facilitates the invasion and metastasis of CRPC cells as shown in vivo xenograft animal experiments.

## Introduction

TGFβ plays many roles during tumor progression. It inhibits epithelial cell proliferation in the early stage but promotes tumor invasion by inducing epithelial-to-mesenchymal transition (EMT) in the late stage [1–3]. Coupled with the strong EMT effect, TGFβ exerts an overwhelming influence on the modification of the extracellular matrix (ECM), which is generally thought to favor the adhesion and migration of tumor cells [4]. However, the underlying molecular mechanism by which the cancer cells take advantage of the ECM environment to facilitate their motility requires further exploration.

TGFβ signals by binding to TGFβ type II receptor (TβRII), which in turn recruits and phosphorylates the TGFβ type I receptor (TβRI) at the glycine–serine rich TTSGSGSG motif, called the GS domain [5]. The activated TβRI phosphorylates and activates the canonic Smad signaling pathway, as well as non-Smad signaling pathways, regulating various cellular events in a context-dependent manner [6–8]. Although the mechanisms regulatingTGFβ signaling have been intensively studied with regard to cell proliferation, survival [9], EMT [3, 4], and cell invasion [10, 11], the function of ECM deposited in the tumor microenvironment by TGFβ signaling has not been elucidated.

The major proteins in the mammalian matrisome are collagens, glycoproteins, and proteoglycans. Collagens are the most abundant proteins in the ECM, providing the mechanic scaffold and bioactive sites for cell adhesion and migration [12, 13]. Glycoproteins and proteoglycans are composed of carbohydrate chains linked to a protein core, building up a highly viscous matrix environment. They also facilitate the organization of other matrix proteins by binding or sequestering growth factors, cytokines, and divalent cations due to their polyanionic charge, creating a cohesive network for the ECM environment [12, 14].

Thrombospondin 1 (THBS1) is a multidomain glycoprotein and a key component of the ECM. It was first discovered in platelets and has an inhibitory effect on angiogenesis [15, 16] but, more recently, it was implicated in tumor development [17]. In glioblastoma, THBS1 is upregulated in high-grade gliomas and associated with poor prognosis [18]. In prostate cancer, THBS1 has been reported to trigger cell migration and the development of advanced prostate tumors [17]. However, how extracellular signals direct the tumor cells to deposit THBS1 in the ECM and how tumor cells exploit the tumor microenvironment to promote malignant transformation are unanswered questions.

In this study, we analyzed the TGFβ signature in prostate cancer and other cancer cells and confirmed that ECM modification and ECM–receptor interaction are the main consequences of TGFβ stimulation. In a mass spectrometry-based secretome analysis, THBS1 was one of the dominant proteins induced by TGFβ and released into the ECM by tumor cells. We further confirmed that TGFβ-induced THBS1 interacts with integrin receptor ITGAV, facilitating the migration and invasion of cancer cells in both in vitro and in vivo models. Interestingly, we observed that TβRI forms a complex with THBS1. Finally, we found that the high expression of THBS1 significantly correlates with the Gleason score (GS), tumor stage, and bone metastasis in prostate cancer. Thus, this study increases knowledge about the oncogenic effect of TGFβ via induction of ECM proteins.

## Results

### TGFβ signaling predominantly regulates ECM proteins

**Fig. 1:**
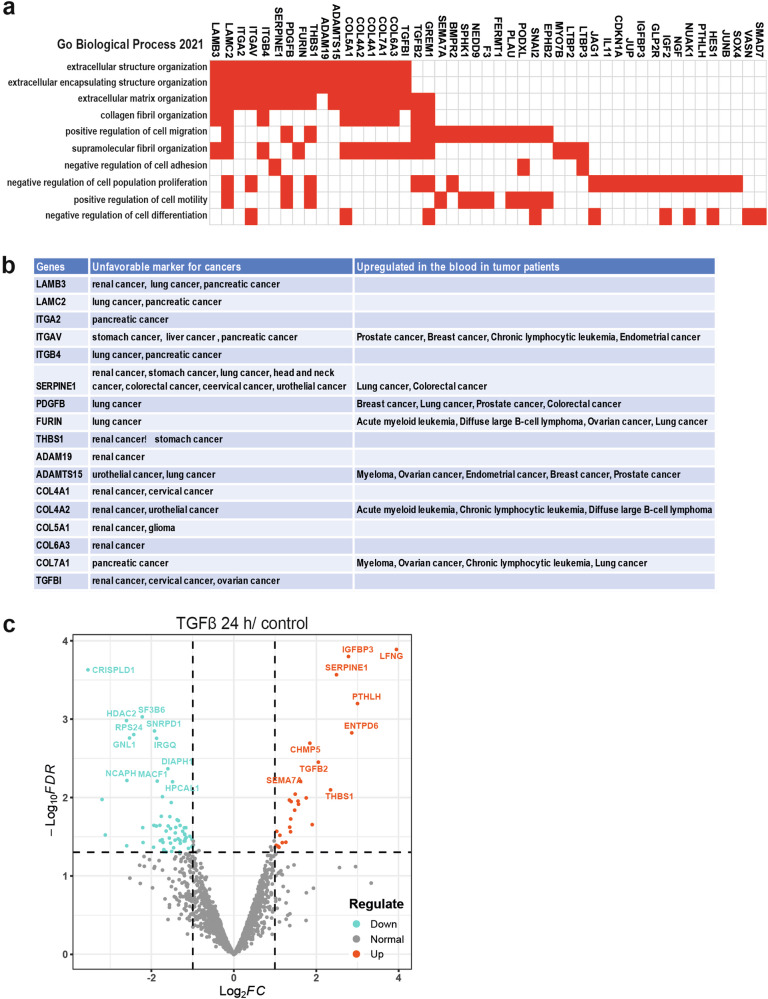
TGFβ signaling predominantly regulates ECM proteins. **a** GO enrichment analysis of the top genes upregulated by TGFβ treatment (fold change > 2) in PC3U cells. **b** The upregulated ECM genes via TGFβ signaling collected from the database of the Human Protein Atlas as prognostic markers for different types of tumors. **c** Mass spectrometry-based secretome analysis showed the differentially secreted proteins in PC3U cells upon TGFβ treatment.

To evaluate the function of TGFβ stimulation on ECM modification in cancer, and to investigate its importance with respect to all of the regulatory functions of TGFβ, we performed a transcriptomic analysis using the human prostate cancer PC3U cell line and the human A549 lung carcinoma cell line. The top-upregulated genes in PC3U cells and A549 cells upon TGFβ stimulation were identified to analyze the main regulatory function elicited by TGFβ signaling. The Gene Ontology (GO) enrichment analysis showed that ECM organization and the positive regulation of cell migration were the top regulatory functions (Fig. 1a and Supplementary Fig. 1). Among the gene products, the laminin protein, integrin, SERPINE1, and THBS1 are known to function in ECM modification and are coupled with positive regulation of cell migration. Notably, most of these TGFβ regulated matrix proteins are markers of poor prognosis in different types of cancers, and some of them are also upregulated in the blood of tumor patients (Fig. 1b) according to the database of the Human Protein Atlas [19, 20]. These data indicate that TGFβ regulatory matrix proteins are closely associated with tumor progression and could play essential roles in TGFβ-driven oncogenic functions. Next, we examined these ECM proteins in secretome analysis of the cancer cells by mass spectrometry-based proteomics. The serum-free culture medium was collected from TGFβ-treated or untreated cells and concentrated, and then the secreted protein was analyzed by mass spectrometry. Interestingly, TGFβ treatment of human castration-resistant prostate cancer (CRPC) (PC3U) cells induced the secretion of multiple proteins (Fig. 1c) that were also consistently detected in our transcriptomic analysis. THBS1, also named TSP1, was recently identified as a non-invasive specific biomarker in the blood of CRPC patients [21]. Therefore, we focused on THBS1 in this study.

### Generation of a TβRI-deficient cell line using CRISPR-Cas9 technology

**Fig. 2:**
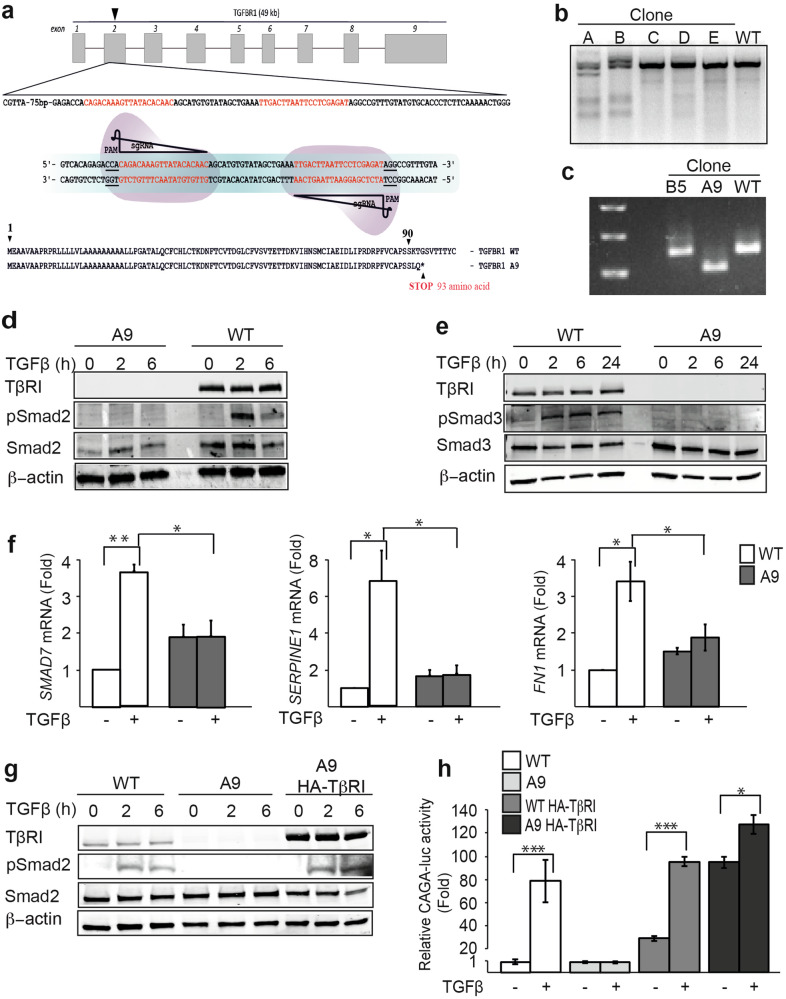
Generation of the TβRI-deficient cell line using CRISPR-Cas9 technology. **a** Exon 2 of TβRI was designed to be targeted by the mutant Cas9 enzyme. The mutant clone A9 contained a deletion of TβRI as detected by sequencing and the stop sequence formed by a frameshift mutation. **b** The mutant cell clones (A–E) and wild-type (WT) cells were examined by Cel-1 assay (Surveyor nuclease assay). **c** Mutant clones B5 and A9 and WT cells detected by RT-PCR. **d**, **e** Western blot and **f** qPCR were used to verify the TβRI-deficient cell lines. Results are shown as mean ± SEM, *p < 0.05, **p < 0.01, Student’s *t*-test. **g** WT, A9, and HA**-**TβR1 reconstituted A9 cells were lysed and subjected to immunoblotting with antibodies TβRI, pSmad2, Smad2, and actin. **h** CAGA-luciferase reporter assay showing the TGFβ response of the indicated cell lines. Results are shown as mean ± SEM, *p < 0.05, ***p < 0.001, Student’s *t*-test.

The CRISPR-Cas9 system has been widely used in molecular studies, enabling the characterization of molecular function both in vitro and in vivo. To further study the regulatory function of TGFβ signaling in ECM modification, we deleted TβRI in PC3U cells using CRISPR-Cas9 technology. Exon 2 of TβRI was targeted by a mutant Cas9 enzyme that minimized the off-target effects (Fig. 2a) [22]. After cloning the two guide RNAs (gRNAs) into Cas9 plasmids, the constructs were transfected into PC3U cells. Positive cells were selected with puromycin and the single-cell clones propagated. PCR amplification and RT-PCR of the region containing the target sequence revealed that several clones had been mutated in exon 2 by the Cas9 enzyme (Fig. 2b, c). Transcription from the region resulted in a shorter product in clone A than the wild-type. The sequencing of the PCR product showed that the Cas9 enzyme and subsequent DNA repair machinery had generated a 44-nucleotide deletion in exon 2 of clone A. This deletion gave rise to a frameshift mutation, resulting in a downstream stop codon (Fig. 2a). Western blot analysis showed that expression of TβRI protein was completely lost in clone A9. The downstream phospho-Smad2 and phospho-Smad3 signaling revealed robust downregulation in the mutant cells compared to the wild-type (Fig. 2d, e). The expression of classical TGFβ target genes, such as *Smad7*, *SERPINE1*, and *FN1*, was also downregulated in the A9 clone (Fig. 2f). In addition, we re-introduced an HA-tagged TβRI (HA-ca ALK5) into A9 cells and found that reconstituted HA-ca ALK5 rescued pSmad2 signaling, which was confirmed by both western blot and CAGA-luciferase reporter assay (Fig. 2g, h). These results demonstrate that, by using Cas9 technology, we obtained a TβRI-deleted cell line in which the TGFβ signals were blocked.

### Blocking of TGFβ signaling impairs the expression of ECM proteins

**Fig. 3:**
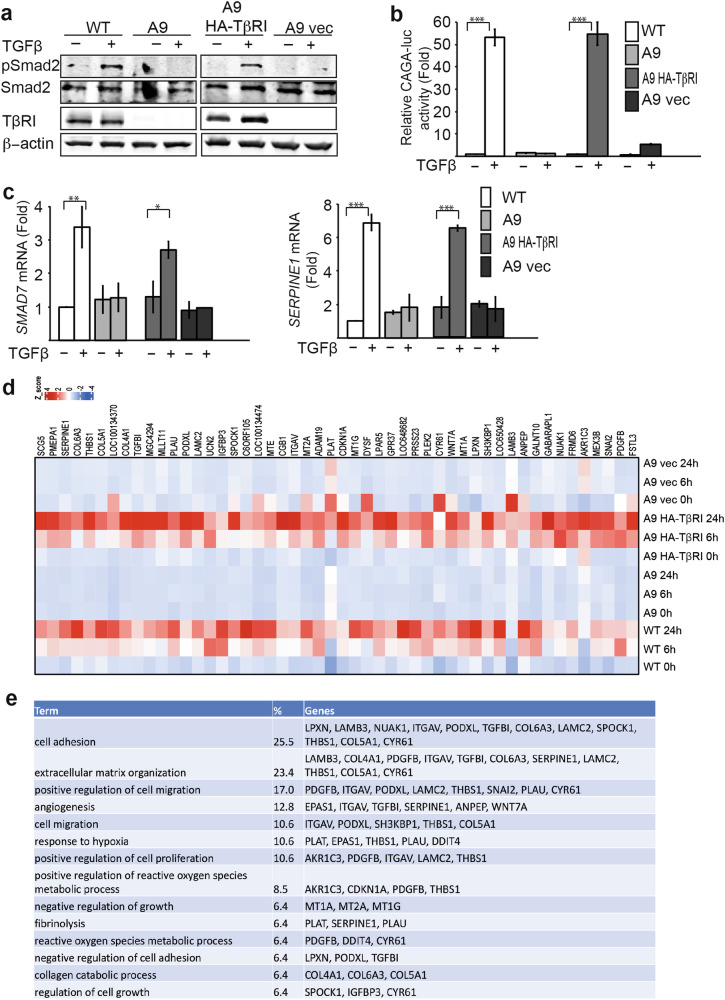
Blocking TGFβ signaling impairs ECM protein expression. **a** Gene-modified cell lines wild-type (WT), A9 (TβRI deficient), A9 HA-TβRI (A9 cells reconstituted with HA-TβRI), and A9 vector (empty vector as control) were subjected to immunoblotting with antibodies pSmad2, Smad2, TβRI, and actin. **b** CAGA-luciferase reporter assay showing the TGFβ response in the indicated cell lines. Results are shown as mean ± SEM, ***p < 0.001, Student’s *t*-test. **c** Expression of *Smad7* and *SERPINE1* in the indicated cell lines detected by qPCR. Results are shown as mean ± SEM, ***p < 0.001, Student’s *t*-test. **d** Microarray data showing the differentially expressed genes (DEGs) in the indicated cell lines with or without TGFβ treatment. **e** DEGs were annotated to the biological functions by DAVID Bioinformatics Resources. *p < 0.05, **p < 0.01.

In A9 cells, we stably expressed HA-tagged TβRI or empty plasmid. These cell lines were examined by western blot analysis, CAGA-luciferase reporter assay (Fig. 3a, b), as well as the TGFβ target genes Smad7 and SERPINE1 (Fig. 3c). We performed a microarray analysis in these cell lines to detect the genes regulated by TGFβ signals. The heatmap of the top differentially expressed genes (DEGs) showed that A9 cells did not respond to signals and blocked the expression of TGFβ target genes. Apparently, the HA-ALK5 reconstituted cells responded to TGFβ signals and upregulated genes in a similar pattern as the wild-type PC3U cells (Fig. 3d). Consistently, the genes identified in the transcriptomic analysis and the secretome of TGFβ-stimulated PC3U cells were found among these top genes. These top genes were then used to annotate the biological functions, which suggested that, consistent with our RNA-seq data, the top regulatory genes were related to cell adhesion and ECM organization (Fig. 3e). Interestingly, blocking the TGFβ signals in A9 cells suppressed the expression of ECM proteins THBS1, TGFBI, and ITGAV, which are strongly related to cell adhesion. Taken together, these data indicate that TGFβ plays a major role in the regulation of ECM proteins that couple with cell adhesion and migration.

### TGFβ-induced THBS1 mediates migration and invasion of cancer cells

**Fig. 4:**
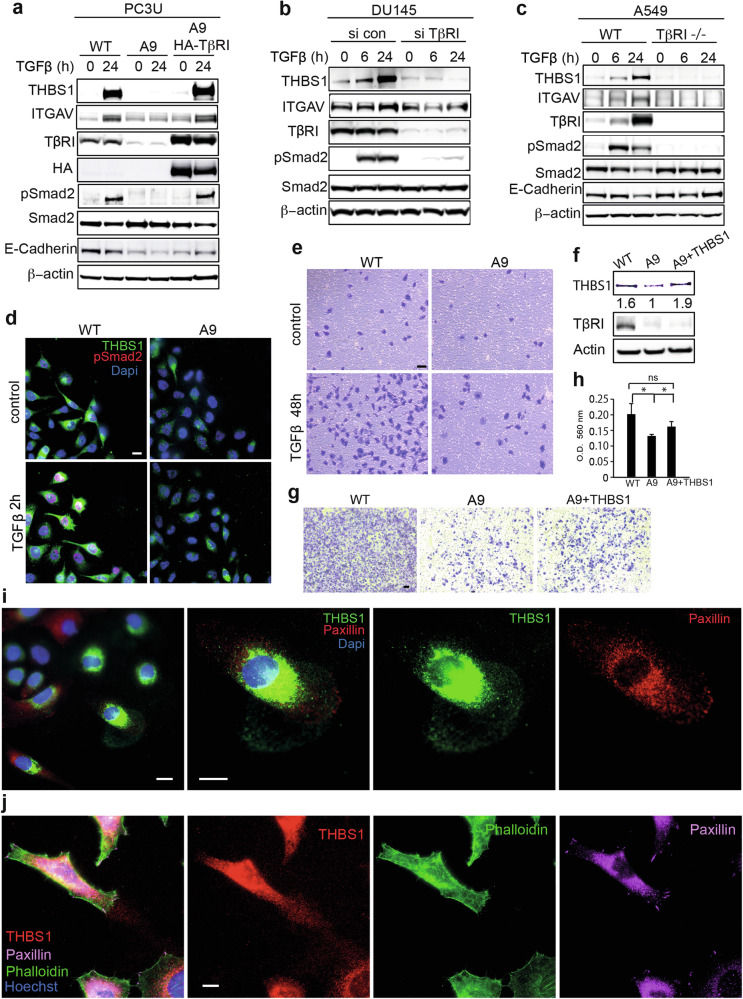
TGFβ-induced THBS1 mediates the migration and invasion of cancer cells. **a**–**c** Expression of THBS1 and ITGAV detected by western blotting in the PC3U, DU-145, and A549 cell lines with or without a knockout or knockdown of TβRI. **d** Immunofluorescent (IF) staining of THBS1 and pSmad2 in WT and A9 cells in a wound-healing assay with or without TGFβ treatment. Scale bar, 20 μm. **e** Invasion assay showing the invasive capacity of PC3U cells with or without knockout of TβRI or with or without TGFβ treatment for 48 h. Scale bar, 50 μm. **f** Western blotting showing the overexpression of THBS1 in A9 cells. **g** Invasion assay showing the invasive capacity of WT, A9, and A9 cells overexpressed THBS1. Scale bar, 50 μm. **h** The invading cells were quantified by OD value. Shown as mean ± SD, *p < 0.05. **i** IF staining of THBS1 and Paxillin in PC3U cells treated with TGFβ for 6 h. Scale bar, 20 μm. **j** IF staining of THBS1, Paxillin, and Phalloidin in PC3U cells treated with TGFβ for 6 h. Scale bar, 20 μm.

ECM remodeling is a critical event in the tumor microenvironment that promotes tumorigenesis and metastasis [23]. A large group of matrix proteins is involved in creating a dynamic environment to facilitate cell–matrix interactions. In our secretome analysis of TGFβ stimulated tumor cells, THBS1 was detected as one of the most abundant proteins. THBS1 is known to activate latent TGFβ, its fundamental role as a viscous glycoprotein has been rarely elucidated. Here, we found that THBS1 was remarkably induced upon TGFβ treatment for 24 h. ITGAV, another ECM protein, functions as a binding receptor for THBS1 and was also induced by TGFβ (Fig. 4a). Interestingly, blocking TGFβ signaling in A9 cells completely prevented this induction. Similar results were observed when TβRI was knocked down transiently with siRNA in DU-145 cells (Fig. 4b) and another TβRI-deleted A549 cell line (Fig. 4c). PC3U cells were originally derived from PC3 cells and express more TGFβ receptors than PC3 originally described in Franzén et al. [24]. DU-145 cells were derived from the brain metastasis of prostate cancer, and A549 cells are lung cancer cells, expressing a high level of TβRI and widely used for TGFβ signaling research (Fig. 4c). These cancer cell lines respond to TGFβ treatment with increased migration and invasion in vitro [25]. Our data including experiments performed in DU-145 and A549 cells suggest that the regulation of THBS1via TGFβ signaling is a general mechanism in aggressive cancer cells. Next, we investigated the motility of cancer cells in a wound-healing assay. Using immunofluorescent staining, we found that TGFβ induced a high level of THBS1 expression in the migratory cells facing toward the wound. When TβRI was deleted, the A9 cells did not respond to TGFβ. Correspondingly, there was a lower level of THBS1 expression and fewer migratory protrusions formed (Fig. 4d). Accordingly, the invasive capacity of A9 cells in response to TGFβ was reduced compared to the wild-type cells (Fig. 4e). To investigate if THBS1 mediates the invasive capacity, we transiently overexpressed THBS1 in A9 cells (Fig. 4f). The invasive capacity of A9 cells was partially rescued (Fig. 4g, h). Interestingly, we observed that THBS1 was released into the matrix and formed a “carpet structure” just around the frontier of the migratory cells, and the focal adhesion perfectly anchored into the “viscous carpet” (Fig. 4i). To carefully examine the distribution of THBS1 in the ECM, we used Phalloidin to visualize the cell cytoskeleton. THBS1 was observed localized in the ECM generally, but, predominantly in the front of the migratory cells, and connected to the focal adhesion structure (Fig. 4j). This finding proposes how THBS1, as a matrix protein, facilitated tumor cells’ migration. In summary, these data indicate that TGFβ-induced THBS1 and other ECM proteins are involved in the migration and invasion of cancer cells in a TβRI-dependent manner.

### THBS1 mediates the cell migration by forming a complex with TβRI and ITGAV

**Fig. 5:**
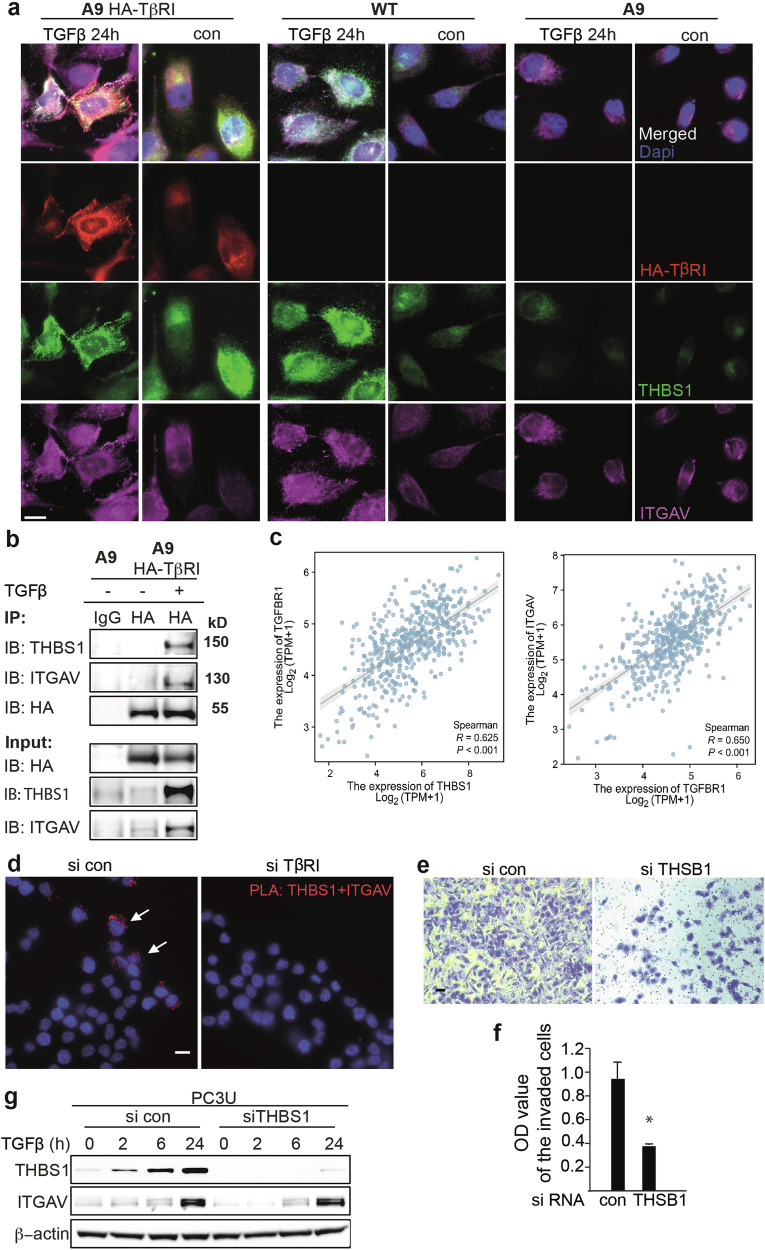
THBS1 mediates the cell migration by forming a complex with TβRI and ITGAV. **a** Co-IF staining of HA, THBS1, and ITGAV in the wild-type (WT), A9 (TβRI deficient), A9 HA-TβRI (A9 cells reconstituted with HA-TβRI) cell lines with or without TGFβ treatment. Scale bar, 20 μm. **b** Co-IP was performed to detect the interaction of HA-TβRI with THBS1 and ITGAV. **c** The correlation of TβRI and THBS1 expression, TβRI and ITGAV expression in prostate cancer (TCGA database). **d** The interaction of THBS1 and ITGAV was detected in a wound-healing assay by in situ PLA. Scale bar, 50 μm. **e** Invasion assay showing that knockdown of THSB1 prevented the invasion capacity of PC3U cells treated with TGFβ. Scale bar, 50 μm. **f** The invading cells were quantified by OD value. Shown as mean ± SD, *p < 0.05. **g** Western blot showing the expression of THSB1 and ITGAV in PC3U cells with THSB1 knockdown.

THBS1 has been reported to be a key regulator of the ECM by interacting with a group of matrix proteins, such as collagen, integrin receptors, and MMPs [18]. We focused on ITGAV because it was upregulated concomitantly with the induction of THBS1 in the cells treated with TGFβ1 (Fig. 4a–c). ITGAV plays an important role in cell adhesion and migration; therefore, we investigated the interaction of THBS1 with ITGAV. In co-immunofluorescence, we observed colocalization of THBS1 and ITGAV upon TGFβ stimulation (Fig. 5a). As known, THBS1 directly binds to the latency-associated peptide (LAP), and ITGAV binds to the Arg-Gly-ASP (RGD) motif of LAP, contributing to the release of active TGFβ [16]. Interestingly, we also found that THBS1 and ITGAV co-localized with TβRI in the migratory ruffling (Fig. 5a), indicating TβRI linked the THBS1 and ITGAV to form a migratory complex. The focal adhesion marker Paxillin was also found co-localized with THBS1 and TβRI in the migratory complex (Supplementary Fig. 2). This colocalization is easily observed in the cell membrane, although three portions of THBS1could be observed according to its distribution, in the ECM, in the cell membrane, or the intracellular compartment, which indicates that THBS1 may play multiple roles in the TGFβ signaling response. We further performed a co-immunoprecipitation (co-IP) experiment in cells stimulated by TGFβ and precipitated HA-tagged TβRI from our gene-modified cell lines using HA-specific antibodies. As expected, TβRI interacted with THBS1 as well as ITGAV, which is TGFβ-dependent (Fig. 5b). This is also consistent with the co-immunofluorescent staining (Fig. 5a). Interestingly, in TCGA database, the expression of TβRI and THBS1, as well as TβRI and ITGAV in prostate cancer shows the correlations (Fig. 5c). Furthermore, we employed proximity ligation assay (PLA) to investigate the interaction of THBS1 with ITGAV in a wound-healing assay. This showed that the interaction of THBS1 with ITGAV frequently appeared in the migratory cells toward the wound, while knockdown of TβRI prevented the formation of the migratory complex (Fig. 5d). These data indicate that TβRI links with the migratory complex comprising the main components THBS1 and ITGAV, to facilitate the cell migration.

To further analyze the function of THBS1 in the migration and invasion of prostate cancer cells, we knocked down THBS1 in PC3U cells using siRNA. In the invasion assay, the knockdown of THBS1 prevented the invasion of prostate cancer cells (Fig. 5e, f). Using immunoblotting, we observed that the regulation of ITGAV by TGFβ was not affected by the knockdown of THBS1 (Fig. 5g), suggesting that the induction of ITGAV was not dependent on THBS1. Taken together, these data indicate that THBS1 is a crucial matrix molecule that mediates tumor cell migration through interaction with adhesion receptor ITGAV.

### A high level of THBS1 is associated with prostate cancer malignancy

**Fig. 6:**
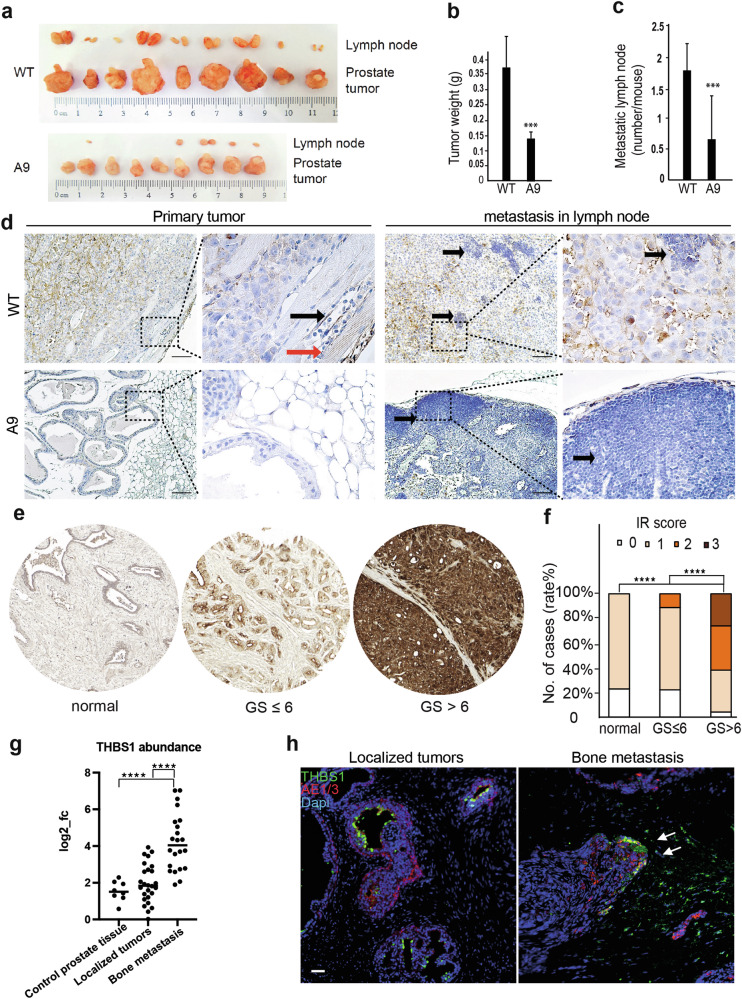
A high level of THBS1 is associated with prostate cancer malignancy. **a** Wild-type (WT) PC3U and TβRI-deficient A9 cells were injected into the prostates of mice. Tumors and sentinel lymph nodes were obtained from the orthotopic xenograft mouse model after 4 weeks. **b** The tumor weight was quantified in both the WT (*n* = 9) and A9 (*n* = 9) groups. Results are shown as mean ± SEM, ***p < 0.001, one-way ANOVA. **c** The metastatic lymph nodes were quantified in both the WT (*n* = 9) and A9 (*n* = 6) groups. Results are shown as mean ± SEM, ***p < 0.001, one-way ANOVA. **d** Representative images of THBS1 staining in the WT or A9 tumors and lymph metastasis. Scale bar, 500 μm. Left: the black arrow shows the lymph vessels in the tumor-invasive front, and the red arrow shows tumor cells infiltrating into the adjacent muscles. Right: the black arrows show the remaining lymphatic cells in the sentinel lymph nodes. Scale bar, 100 μm. **e** Representative images of THBS1 staining in the normal adjacent prostate tissue, lower malignant prostatic adenocarcinoma (GS ≤ 6), and higher malignant prostatic adenocarcinoma (GS > 6). **f** THBS1 expression was examined on tissue microarray sections with 192 samples. Immunoreactivity (IR) was scored as negative (IR = 0), weak (IR = 1), moderate (IR = 2), or strong (IR = 3) ****p < 0.0001, Tukey’s multiple comparisons test. **g** A proteomic database was used to analyze the THBS1 protein level in the control adjacent prostate tissue (*n* = 8), localized tumor (*n* = 28), and bone metastasis (*n* = 22). ****p < 0.0001, one-way ANOVA. **h** Representative images for the IF staining of THBS1 and panCK AE1/3 in the human primary and bone metastatic tumors. Scale bar, 50 μm.

To further investigate the role of THBS1 in tumor invasion and metastasis, we applied an orthotopic human metastatic CRPC xenograft model that was previously established in our laboratory [25] to elucidate the molecular mechanisms regulating lymphatic metastasis of prostate cancer. We used the wild-type PC3U cells and TβRI-deleted A9 cells to analyze the invasion and metastasis of cancer cells in vivo. Compared to the wild-type PC3U cells, which formed a large tumor and disseminated into the adjacent lymph nodes, the A9 cells formed a smaller tumor with fewer lymph metastases (Fig. 6a–c). Interestingly, we observed that A9 cells reconstituted with HA-TβR1 partially rescued their non-invasive phenotype in vivo (Supplementary Fig. 3a, b). The expression of THBS1 was examined by immunohistochemistry staining and a higher level of THBS1 was observed in the wild-type tumors and their lymph metastases compared with the A9 tumors (Fig. 6d). This indicates that blocking TGFβ signaling inhibited the production of THBS1 and the invasion of prostate cancer cells. Since THBS1 has an anti-angiogenic role, it is interesting to observe the angiogenesis in the xenografted tumors. We stained the vascular marker CD31 and quantified the blood vessels. The density of the blood vessels was higher in the WT tumors than in the A9 tumors (Supplementary Fig. 3c). We thought this might be due to the strong pro-angiogenesis effect via TGFβ signaling that must be independent of the activity of thrombospondin. We also investigated THBS1 in human prostate cancer tissue using immunohistochemical staining. The tissue microarray sections, including 192 clinical samples, were used to evaluate the expression of THBS1 by the immunoreactivity and GS (representative images in Fig. 6e). GS > 6 represented more aggressive tumors, and these exhibited increased expression of THBS1 (Fig. 6f). This was significantly higher than in the adjacent normal prostate tissue or tissue with less malignancy (GS ≤ 6). We used a published proteomic database that includes proteomic profiling of 8 normal prostate tissues as control, 28 localized prostate tumors, and 22 bone metastases [26] and found that the expression of THBS1 was significantly higher than in the control prostate tissue and localized prostate tumors (Fig. 6g). In addition, immunofluorescent staining indicated a higher level of THBS1 in bone metastatic prostate cancer. THBS1 was deposited in the frontier of metastatic tumor cells, but this was not obvious in the primary tumors (Fig. 6h). These findings indicate that the TGFβ-induced ECM protein THBS1 was associated with the malignant phenotype and bone metastasis of prostate cancer.

## Discussion

ECM remodeling is the fundamental step promoting tumor cell migration and invasion, and recent knowledge suggests that the ECM organization and composition, in addition to density and stiffness, is supporting tumor cell motility and tumor progression [23]. As non-stiffness proteins, the glycoproteins and proteoglycans accumulate in the tumor matrix to create a highly dynamic microenvironment for tumor cells. In this study, we found that THBS1 is highly expressed and secreted by PC3U cells, aggressive human CRPC cells in vitro, as well as in clinical materials. As a large glycoprotein, THBS1 was previously identified to be an activator of latent TGFβ and an endogenous inhibitor of angiogenesis [15]. More recently, THBS1 was recognized as a central regulator of glioblastoma invasiveness, as a bioinformatics analysis predicted its high connectivity with collagens, TGFβ1, and integrins [18]. In a study from Wang et al. [27], performed in DU-145 and PC3 prostate cancer cells, the authors demonstrated an opposite role for THBS1, as methyltransferase-like14 by epigenetic modulation downregulated THBS1 expression, leading to an increase of proliferation of the prostate cancer cells. THBS1 is a multifunctional protein, and one of its functions is to cause activation of latent TGFβ [16]. In our study we observed that TGFβ treatment of PC3U cells resulted in increased secretion of THBS1 which promoted migration and invasion of cancer cells. The distinct function of THBS1 acting as a tumor promoter in our study, while THBS1 inhibited the proliferation of PC3 and DU-145 cells, as reported by Wang et al. [27], might be related to the dual activities of TGFβ in cancer. TGFβ acts as a tumor suppressor at early stages, but tumor promoting once the negative effects of TGFβ on proliferation are curtailed by molecular events that drive tumor progression [1]. As we also observed a significant increase of THBS1 in clinical prostate cancer tissues, with higher GS (Fig. 6e, f) and increased abundance of THBS1 in localized tumors, and bone metastases (Fig. 6g), our data are in line with the previous observation that THBS1 can promote tumor aggressiveness in advanced prostate cancer [17].

Interestingly, we observed that THBS1 was specifically deposited in the matrix at the migratory front of the cell, where it seems to pave the way for cancer cells to migrate. However, the tumor matrix protein is highly dynamic and undergoes continuous turnover, so it is generally not easy to study matrix proteins. We expect that advances in molecular approaches and imaging techniques will enable even more specific studies to uncover the functions of the tumor matrix in the highly dynamic tumor microenvironment.

Orchestration of the ECM structure primarily depends on external signaling, and TGFβ is thought to be important in triggering both stromal cells and cancer cells to contribute to both matrix deposition and remodeling [23]. As confirmed by our transcriptome analysis, TGFβ-regulated genes encoding proteins that are predominantly involved in the ECM structure, promotes cell migration. These data emphasize the major function of TGFβ in ECM remodeling, which may be a crucial part of the fundamental regulatory mechanism driving cancer cells to a malignant transition. Although a previous study showed that THBS1 is associated with the development of advanced prostate cancer [17], the molecular function of THBS1 in the context of prostate cancer progression has previously not been uncovered. Moreover, here, we verified that THBS1 is the main target gene via TGFβ signaling and one of the main proteins detected in a secretome analysis of human CRPC cells. We further confirmed that TGFβ1-induced THBS1 is important for cancer cell invasion, as the knockdown of THBS1 in vitro, or knockdown of TβRI both in vitro and in vivo, inhibited the invasion of prostate cancer cells.

THBS1 is known as an adhesive protein that mediates cell–cell contacts and cell–matrix contacts, but the function of THBS1 in the regulation of cell migration has rarely been studied. Here, we applied gene-modified cell lines and detected the interaction of THBS1 with TβRI and ITGAV. Binding to adhesion receptor ITGAV is an important step for cell migration, which was confirmed by a PLA assay. We did observe more protein complexes appearing in the migratory cancer cells, and THBS1 functions as an important mediator with TβRI and ITGAV. ITGAV is also one of the TGFβ-regulated genes, as we observed in the different cancer cell lines by western blot, immunofluorescence, and RNA-seq. Interestingly, both THBS1 and ITGAV are key players for activation of TGFβ, by releasing it from the large latent complexes in which it is secreted and deposited in the ECM [28]. As we observe that TβRI is forming a protein complex with THBS1 and ITGAV in the frontier of the migratory PC3U cells, we think it might be a way for the identified protein complex to facilitate the localization of active TGFβ close to the receptors, as both THBS1 and ITGAV can facilitate the release of active TGFβ from its latent complex.

Except for integrins, other matrix proteins regulated by TGFβ were confirmed in our study, including the collagen family, laminin, and other proteoglycans and matrix proteinases. Most of these proteins are poor prognostic biomarkers for different types of cancers [29], and many are upregulated and detectable in the blood derived from patients with cancer. Notably, TGFβ is elevated in prostate cancer tissues and in the circulation of prostate cancer patients [30–32]. These data suggest that TGFβ itself and TGFβ-regulatory ECM proteins could be applied as diagnostic markers for different types of cancers or even be useful as non-invasive biomarkers by analyzing blood samples. Importantly, THBS1 was recently examined in the exosomes of patients with CRPC and identified as a potential non-invasive marker for guiding treatment decisions [21].

In summary, we report here that THBS1 is an abundant ECM protein in aggressive tumors and is induced by TGFβ stimulation of CRPC cells. TGFβ signaling plays a major role in governing the deposition of ECM protein, which promotes cancer cell migration. Notably, we found that TβRI is forming a protein complex with ITGAV and THBS1 in the front of migrating PC3U cells, thereby utilizing the matrix protein THBS1. Thus, we elucidated a novel oncogenic role of TβRI in TGFβ signaling in the context of cancer cell migration, invasion, and metastasis.

## Materials and methods

### Cell lines

Human prostate cancer PC3U cells are a subclone of PC3 cells, and they express more TGFβ receptors than PC3 cells, originally described in Franzén et al. [24]. PC3 is a cell line initiated from a bone metastasis of a grade IV prostatic adenocarcinoma from a 62-year-old, White, male. PC3U cells are more invasive than PC3 cells, and the similarities between PC3 and PC3U cells have been examined by whole genome sequencing reported in Zang et al. [25]. Human prostate cancer DU-145 cells which are derived from the brain metastasis of prostate cancer were purchased from ATCC, Cat. 30-2003. Human lung cancer cell line A549 was purchased from Sigma, Cat. R0883.

### Cell culture

The human prostate cancer cell line PC3U and human lung cancer cell line A549 were grown in RPMI-1640 (Sigma, Cat. R0883) with l-glutamine (Sigma, Cat. G7513), and DU-145 was grown in EMEM (ATCC, Cat. 30-2003) supplemented with 10% fetal bovine serum (FBS) (Sigma, Cat. F7524). The cells were starved for 12–18 h in a medium supplemented with 1% FBS before stimulation with TGFβ1 (Prospec, Ness-Ziona, Israel) at a concentration of 10 ng/mL. All experiments were performed with mycoplasma-free cells.

### Antibodies and reagents

The antibody against TβRI (Cat. SC-398) was from Santa Cruz, and the rabbit monoclonal antibody against TβRI (Cat. 235578) was from Abcam (Cambridge, UK) its specificity was verified by knockout cells. The antibodies against HA (Cat. 2367S), pSmad2 (Cat. 3108L), Smad2 (Cat. 3103S), and pSmad3 (Cat. 9520S) were purchased from Cell Signaling Technology. Antibodies against Smad3 (Cat. ab40854) and ITGAV (Cat. ab179475) were from Abcam, THBS1 (Cat. AF3074) from R&D System, Paxillin (Cat. 10029-1-Ig) and E-cadherin (Cat. 610182) from BD Biosciences, β-actin (Cat. A1978) from Sigma, AE1/AE3 (Cat. M351501-2) from Agilent, Alexa fluor 647 (Cat. A31571) from Invitrogen, and Alexa fluor 488 (Cat. 705-546-147) and Cy3 (Cat. 711-166-152) from Jackson ImmunoResearch. 4,6-Diamidino-2-phenylindole dihydrochloride (DAPI) (Cat. 5087410001) was from Merck. Pefabloc was from Roche (Mannheim, Germany), and PageRuler prestained protein ladder was from Thermo Scientific. Plasmid pGEM2hTSP-1 was a gift from Jack Lawler (Addgene plamid # 12993).

### RNA-seq and bioinformatics analysis

RNA was extracted from PC3U cells or A549 cells treated with or without TGFβ1 using the AllPrep DNA/RNA/Protein Mini Kit (QIAGEN, Cat. 80004) and subjected to RNA-seq at Novogene (Novogene Bioinformatics Institute, Beijing, China). The DEGs were analyzed with support from the Bioinformatics Infrastructure for Life Sciences (BILS). The top DEGs in PC3U cells and A549 cells that were upregulated (fold change > 2) by TGFβ1 treatment were used for GO enrichment analysis in the online tool Enrichr [33–35].

### Secretome analysis

To collect the secreted proteins, the cells were washed two times with PBS and then cultured with TGFβ1 for 24 h in a medium without FBS. The culture media were collected and centrifuged at 1000 × *g* for 10 min to remove the cell debris. The supernatant was carefully removed, concentrated from 50 mL to 500 µL with Amicon Ultra-15 (Sigma, Darmstadt, Germany), and frozen at −20 °C for LC–MS/MS.

Samples were digested into peptides using a modified SP3 protocol [36, 37]. Briefly, samples were denatured in lysis buffer (2% sodium dodecyl sulfate (SDS), 20 mM tris(2-carboxyethyl) phosphine hydrochloride (TCEP)). SpeedBeads (Sigma Aldrich, beads A, Cat. GE45152105050250; beads B, Cat. GE65152105050250) were mixed with each sample and transferred onto a filter plate (Sigma Aldrich, Cat. MSGVN2210). The samples were washed to remove the unbound fraction and then digested overnight at room temperature with shaking. After centrifugation, the flowthrough containing peptides were collected and the bound peptides were eluted from the beads by adding 10 µL 2% DMSO. The obtained fraction was pooled with the previous flowthrough. Peptides were desalted on the Oasis HLB plate (Waters, Cat. 186001828BA) and dried.

Dried peptides were dissolved in 0.1% formic acid, and 1 µg peptide was used to obtain the mass spectrum using Vanquish Neo (Thermo Scientific). Trapping column PEPMAP NEO C18 (Thermo Scientific) and analytical column nano EaseTM M/Z HSS C18 T3 (Waters) were used for LC–MS/MS. Data acquisition was carried out on an Exploris 480 (Thermo Scientific) in a data-dependent method. Raw data were analyzed using FragPipe (version 18), and R (version 4.2.2) was used for statistical analysis and volcano plots. The data were normalized using the vsn package [38], and the differentially expressed proteins were analyzed using the limma package. The differences in protein abundances were statistically determined using the Student’s *t*-test moderated by Benjamini–Hochberg’s method.

### Generation of TβRI-deficient cell line

The CRISPR mCas9 plasmid was from Addgene (PX462) [22], and two gRNA sequences targeting exon 2 of TβRI were chosen: (sense, TTGACTTAATTCCTCGAGAT and antisense, ATCTCGAGGAATTAAGTCAAC; sense, GTTGTGTATAACTTTGTCTG and antisense, CAGACAAAGTTATACACAACC). The complementary oligonucleotides were annealed, phosphorylated, and cloned into the PX462 vector, and the correct insertion of the gRNA was verified by sequencing. The gRNA-mCas9 plasmids were transfected into PC3U cells using xTREMEGene9 DNA transfection reagent (Roche) in 6-well plates. After transfection for 48 h, puromycin was used for selection in 10-cm dishes until the cells grew confluent. Single-cell clones were produced by serial dilution in a 96-well plate and screened to obtain the TβRI-deficient cell line.

### PCR, single-strand conformation polymorphism analysis, and Cel-1 assay

Genomic DNA and total RNA were exacted from cells using ALLprep (Qiagen). The Cas9-targeted region in exon 2 of the TβRI gene was amplified by PCR. As in our previous report [25], PCR products were denatured and loaded on a pre-cast 12.5% acrylamide gel. For the Cel-1 assay (also named Surveyor nuclease assay), PCR products were denatured and re-annealed, digested with SURVEYOR Nuclease (Transgenomic, Omaha, USA), separated on the 10% polyacrylamide gel, and visualized by the silver staining method.

### RT-PCR, qPCR, and primers

Purified total RNA (2 μg) was used as a template for cDNA synthesis in the Thermoscript RT-PCR system (Invitrogen Cat. 11146016) according to the manufacturer’s instructions. Purified cDNA was amplified and measured with the Stratagene RT-PCR system, and SYBR Green (Applied Biosystems Cat. 4385612) was used for qPCR. The primers used were: TβRI, forward primer (FP), GTGACAGATGGGCTCTGCTT, reverse primer (RP), AAGTGCTGCTCTGAATCCCC; Smad7, FP, CATGGTGTGCGGAGGTCAT, RP, GAGCGCAGATCGTTTGGTC; SERPINE1, FP, AGAGCGCTGTCAAGAAGACC, RP, AGTTCTCAGAGGTGCCTTGC; Fibronectin1 (FN1), FP, CCGTGGGCAACTCTGTC, RP, TGCGGCAGTTGTCACAG.

### Sequencing analysis

RNA was purified from A9 cells, reverse transcribed into cDNA, and the Cas9-targeted region PCR-amplified for sequencing. Sequencing PCR (BigDye Terminator v 3.1 cycle sequencing kit) was performed using this amplified PCR product. The sequencing results were analyzed by Sequence Scanner v 1.0 software. Sequencing primers were: FP, GTGACAGATGGGCTCTGCTT; RP, AAGTGCTGCTCTGAATCCCC.

### Reconstitution of HA-tagged T**β**R1 in A9 cells

For reconstitution of cells with C-terminally HA-tagged wild-type TβR1, 5 × 10^5^ mutant PC3U (TβR1-deficent) cells from clone A9 were transfected with plasmid bearing the insert using the xTREMEgene9 DNA transfection reagent. After transfection for 24 h, Geneticin (500 μg/mL) was added to the cells for positive selection of transfected cells. Dead cells were continuously removed, and positive selection was maintained until the cells were confluent, after which the selection with Geneticin was stopped. Stable expression of HA-TβR1 was verified by western blotting the protein extract.

### CAGA-luciferase reporter assay

As reported previously [39, 40], PC3U wild-type and gene-modified cells were transiently transfected with the TGFβ/Smad-responsive promoter CAGA reporter pCAGA12-MLP-luc promoter report for 36 h prior to TGFβ treatment for 16 h. pCMV-β-gal was transfected as a reference and other constructs were included as noted in the figure legends. Luciferase activity was measured in triplicate samples using the enhanced luciferase assay kit following the manufacturer’s protocol (BD Biosciences). Normalized promoter activity data are plotted in bar graphs as the mean ± SEM of at least three independent experiments.

### Microarray analysis

As previously reported [41], total RNA from wild-type, mutant, and reconstituted HA-TβR1 were extracted using ALLprep (Qiagen). After evaluation of RNA quality using the Agilent RNA 6000 Nano Kit and Agilent 2100 Bioanalyzer, 750 ng RNA was used for the preparation of double-stranded cDNAs, and biotinylated cRNAs were hybridized to an Illumina Human HT-12 Beadchip (Illumina) according to the protocol. Microarray data were analyzed using GenomeStudio and DAVID Bioinformatics Resources.

### siRNA transfection

SMART pool siRNA for TβRI or THSB1 and a non-specific control siRNA were purchased by Dharmacon Research (Lafayette, CO). The siRNA was transfected using Oligofectamine (Invitrogen, Carlsbad, CA) according to the manufacturer’s protocol as previously reported [11].

### Immunoblotting and protein interactions

The cultured cells were washed once with ice-cold PBS and lysed in ice-cold lysis buffer (150 mM NaCl, 50 mM Tris pH 8.0, 0.5% (v/v) DOC, 1% (v/v) NP40, 10% (v/v) glycerol, 1 mM aprotinin, 1 mM Pefabloc, and 2 mM sodium orthovanadate). After centrifugation, the supernatant was collected and protein concentrations were measured by a BCA protein measurement kit (Thermo Scientific). An equal amount of protein from the total cell lysate was separated by SDS-PAGE and transferred onto polyvinylidene difluoride membranes. The membranes were blocked for 1 h with 5% BSA in Tris-buffered saline with 0.1% Tween 20 (TBS-T) and then incubated with specific antibodies overnight at 4 °C. The membranes were then washed three times with TBS-T, incubated for 1 h with a secondary horseradish peroxidase-conjugated antibody, and developed with ECL Western blotting detection reagent. Bands were detected using the Amersham Imager RBG system (GE Healthcare, Buckinghamshire, UK).

To detect protein–protein interactions, the cell lysates from HA-TβR1 reconstituted cell lines were subjected to immunoprecipitation with anti-HA antibodies, followed by immunoblotting with THBS1, ITGAV, and HA antibodies.

### Wound-healing assay

Cells grown on sterile, uncoated cover slides in a serum-starved condition were scratched using a 1000 µL pipette tip. After washing with PBS, the cells were treated with TGFβ1 for the indicated time periods, and then fixed for immunofluorescent staining or in situ PLA.

### Immunofluorescence staining of cultured cells

Cells were grown on the sterile glass microscope slides in a 6-well plate under the indicated conditions. The slides were washed four times with PBS, fixed in 4% paraformaldehyde for 30 min at room temperature, washed four times in PBS, and subsequently permeabilized in 0.2% Triton X-100 in PBS for 10 min and blocked in 10 mM glycine overnight. The slides were incubated with the primary antibodies in a humid chamber for 1 h at room temperature. After washing with PBS, the slides were incubated with secondary antibodies labeled with fluorescent dye for 45 min at room temperature, and then with DAPI (Invitrogen, Oregon, USA) for 5 min. Mounting medium was added to the slides for imaging. The samples were analyzed under a fluorescence microscope (Axioplan 2; Carl Zeiss Microimaging, Inc.) with a digital camera (C4742-95; Hamamatsu) using a Plan-neofluar 63× objective lens (Carl Zeiss MicroImaging, Inc.). Primary images were acquired with the camera’s QED software at room temperature.

### In situ proximity ligation assay

In situ PLA was performed according to the protocol for the Duolink PLA probe and Duolink Detection Kit (Sigma). Briefly, cells were fixed on the glass cover, blocked, and incubated with two primary antibodies produced in different species overnight at 4 °C. After washing, the cells were incubated with two sets of probes conjugated with secondary antibodies. The probes were then ligated with a bridging probe in a proximity-dependent manner, which allowed rolling-circle amplification. Finally, the interacted proteins could be visualized as fluorescent dots under the microscope.

### Invasion assay

Invasion assays were carried out in Growth Factor Reduced Corning Matrigel Invasion Chambers (Cell Biolabs, Inc., San Diego, CA). The Matrigel layer of the cell culture inserts was rehydrated in 500 μL serum-free RPMI-1640 media, and 2.5 × 10^4^ cells were seeded into the upper side of the chambers in 1% serum RPMI-1640. The lower wells of the invasion plates were filled with 750 μL RPMI containing 10% FBS. Non-invasive cells were removed from the upper chamber after incubation for 48 h, and the invasive cells were photographed under a Leica DMR light microscope after staining with crystal violet cell stain solution. Colorimetric quantification was performed by transferring inserts into 200 μL of extraction solution for 10 min. The optical density (OD) was measured at 560 nm in a 96-well plate by a Multiskan FC Microplate Photometer (Thermo Scientific).

### Xenograft animal experiments

Male athymic nude mice (Hsd:Athymic Nude- Foxn1^nu^, Harlan) were purchased from Harlan Laboratories, UK (5–6 weeks of age) and maintained at the animal facility at Umeå University. All animal experiments were approved by the Umeå Ethical Review Board in full agreement with the Swedish Ethical Review Act (Approval ID: A26-2022).

As our previous report [25], this orthotopic animal model is reproducible. Briefly, mice were grouped randomly, 2% isoflurane was used to anesthetize the mice and the prostate exposed via an abdominal incision. 1 × 10^5^ wild-type or mutant PC3U cells were injected into the central prostate. The mice were monitored, and body weight was measured twice a week. Following the Swedish Ethical Review Act, the animals will be sacrificed when they show illness or lose weight sharply, otherwise, 4 weeks after the injection is the earliest suitable time to sacrifice the animals. Tumors and sentinel lymph nodes were resected and weighed. Small portions of the tumor tissues were frozen in liquid nitrogen for protein and RNA extraction. The remaining tumor tissues and lymph nodes were fixed in formalin and embedded in paraffin. The morphological evaluation under light microscopy is performed by another researcher as double-blind.

### Immunohistochemistry staining for paraffin-embedded sections

Paraffin-embedded sections were rehydrated in xylene twice for 10 min each; 100% ethanol for 10 min; 95%, 80%, 70% ethanol, and deionized H_2_O for 5 min each; and then in PBS for 10 min. Thereafter, the sections were treated with Antigen Retrieval Reagent (Histolab, Cat. BC-DV2004MX; Cat. HTR1001M) at 95 °C for 5 min and then rinsed with PBS. The sections were kept in 0.75% H_2_O_2_/75% methanol for 30 min, washed twice with PBS, and blocked in 5% normal goat serum for 1 h at room temperature. The sections were incubated with primary antibodies overnight at 4 °C. After being washed with PBS three times for 5 min each, the sections were incubated with a secondary antibody (DAKO Envision system, Denmark) for 45 min at room temperature, followed by three washes in PBS. The sections were then developed using the Betazoid DAB Chromogen Kit (Histolab, Cat. BDB2004L), counterstained with hematoxylin, and mounted in an aqueous mounting medium (Vector Laboratories). Digital images were acquired by scanning with Pannoramic 250 Flash II (3DHistech, Hungary) and analyzed by ImageJ software. Human tissue microarray sections (Cat. PR1921c, PR208a) were purchased from TissueArray, US Biomax.

### Immunofluorescent staining for paraffin-embedded sections

The paraffin-embedded sections with prostate cancer were kindly offered by the Department of Medical Bioscience, Pathology at Umeå University Hospital. The Umeå Ethical Review Board granted ethical permits to use decoded, tumor tissues to generate tissue slides in full agreement with the Swedish Ethical Review Act. As described above, the sections were rehydrated, retrieved, and blocked, and then were incubated with primary antibodies overnight at 4 °C and secondary antibodies for 45 min at room temperature. Finally, the sections were washed with PBS three times each for 5 min, mounted with mounting media with DAPI, and visualized with a fluorescence microscope (Axioplan 2; Carl Zeiss Microimaging, Inc.).

### Quantitative assessment

The digital images were first scored by the proportion of stained cells multiplied by the intensity of staining. The proportion of stained cells was graded into six levels (1, 0–4%; 2, 5–19%; 3, 20–39%; 4, 40–59%; 5, 60–79%; 6, 80–100%), and the intensity of staining was graded into four levels (0, negative; 1, weak; 2, intermediate; 3, strong). Finally, THSB1 immunoreactivity was scored as four grades (grade 0, 0; grade 1, 1–6; grade 2, 7–12; grade 3, 13–18). Tukey’s multiple comparisons test was performed for statistical analysis.

### Statistical analysis

Two-tailed unpaired Student’s *t*-test was performed for the two groups comparison with normal distribution. The Mann–Whitney *U* test was used for non-parametric data. *p* ≤ 0.05 was considered statistically significant. Error bars represent the standard deviation (SD) of the mean, unless otherwise is indicated. Statistical tests and the number of repeats are described in the figure legends.

## Supplementary information

Supplemental Material Mu et al. Landström

## Data Availability

The data generated in this study are available within the article and its supplementary information files. The raw data generated in this study are publicly available in Gene Expression Omnibus (GEO) at GEO accession GSE274287, https://www.ncbi.nlm.nih.gov/geo/query/acc.cgi?acc=GSE274287 (Token edctckgafdqhbit).

## Materials availability

The materials generated and analyzed during the current study are available from Marene Landström, on reasonable request.
