# Supplementary material for: The TβRI promotes migration and metastasis through thrombospondin 1 and ITGAV in prostate cancer cells
Source: Oncogene. 2024 Sep 20;43(45):3321–34. doi: 10.1038/s41388-024-03165-3 (PMC11534692; doi:10.1038/s41388-024-03165-3)
Supplement: Supplementary file 1 — Supplemental Material Mu et al. Landström [file 41388_2024_3165_MOESM1_ESM.pdf]

The TβRI promotes migration and metastasis through thrombospondin 1 and ITGAV in prostate cancer cells

Yabing Mu, Anders Wallenius, Guangxiang Zang, Shaochun Zhu, Stina Rudolfsson, Karthik Aripaka, Anders Bergh, André Mateus, Maréne Landström

Supplementary figures: S1-S3

term (Go Biological Process 2021)	p-value	overlap_genes
extracellular structure organization	2.492183e-17	[COL27A1, MMP2, ITGA2, COL22A1, SERPINE1, PDGFB, LAMC2, FURIN, FGF2, THBS1, TGFBR1, COL1A1, ADAM19, ADAMTS15, COL5A1, SH3PXD2A, COL4A2, COL4A1, ITGA11, TGFBI, ITGA5]
extracellular matrix organization	1.351153e-16	COL1A1, ADAM19, ADAMTS15, COL5A1, SH3PXD2A, COL4A2, COL4A1, P4HA3, ITGA11, TIMP2, TGFBI, ITGA5]
external encapsulating structure organization	4.270467e-16	[COL27A1, MMP2, ITGA2, COL22A1, SERPINE1, PDGFB, LAMC2, FURIN, FGF2, THBS1, COL1A1, ADAM19, ADAMTS15, COL5A1, SH3PXD2A, COL4A2, COL4A1, ITGA11, TGFBI, ITGA5]
regulation of cell migration	5.764716e-11	[SEMA7A, WNT5B, SERPINE2, SPHK1, SERPINE1, TPM1, PDGFB, PIK3CD, LAMC2, FGF2, RHOD, THBS1, TGFBR1, SMAD7, VEGFA, COL1A1, CLDN4, PODXL, CCN1, EPHB2]
positive regulation of cell motility	1.930044e-10	[SEMA7A, WNT5B, SPHK1, PDGFB, PIK3CD, LAMC2, RHOD, THBS1, TGFBR1, VEGFA, COL1A1, CLDN4, PODXL, CCN1, EPHB2]
positive regulation of cell migration	3.291551e-10	[SEMA7A, BMPR2, WNT5B, SPHK1, PDGFB, PIK3CD, LAMC2, RHOD, THBS1, TGFBR1, VEGFA, COL1A1, CLDN4, PODXL, CCN1, EPHB2]
positive regulation of angiogenesis	2.356922e-08	[JUP, SPHK1, SERPINE1, PIK3CD, ANGPTL4, ITGA5, FGF2, THBS1, RUNX1, VEGFA]
collagen fibril organization	2.992008e-08	[COL1A1, COL27A1, COL4A2, COL5A1, COL4A1, COL22A1, P4HA3, LAMC2, TGFBR1]
regulation of cell adhesion	8.714053e-08	[ADGRG1, NUAK1, PODXL, TPM1, TGFBI, EPHB2, RHOD, TGM2, RND1, VEGFA]
positive regulation of vasculature development	9.897607e-08	[JUP, SPHK1, SERPINE1, PIK3CD, ANGPTL4, FGF2, THBS1, RUNX1, VEGFA]

Fig. S1. TGFβ signaling predominantly regulates ECM proteins in A549 cells. GO enrichment analysis of the top genes upregulated by TGFβ treatment (fold change>2) in A549 cells.

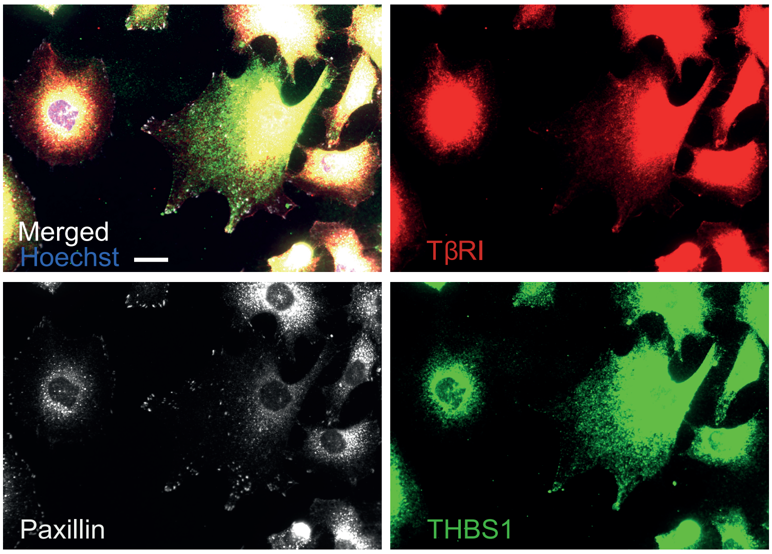

Fig. S2. IF staining shows the co-localization of TβR1, Paxillin, and THBS1 in the migratory complex in PC3U cells.

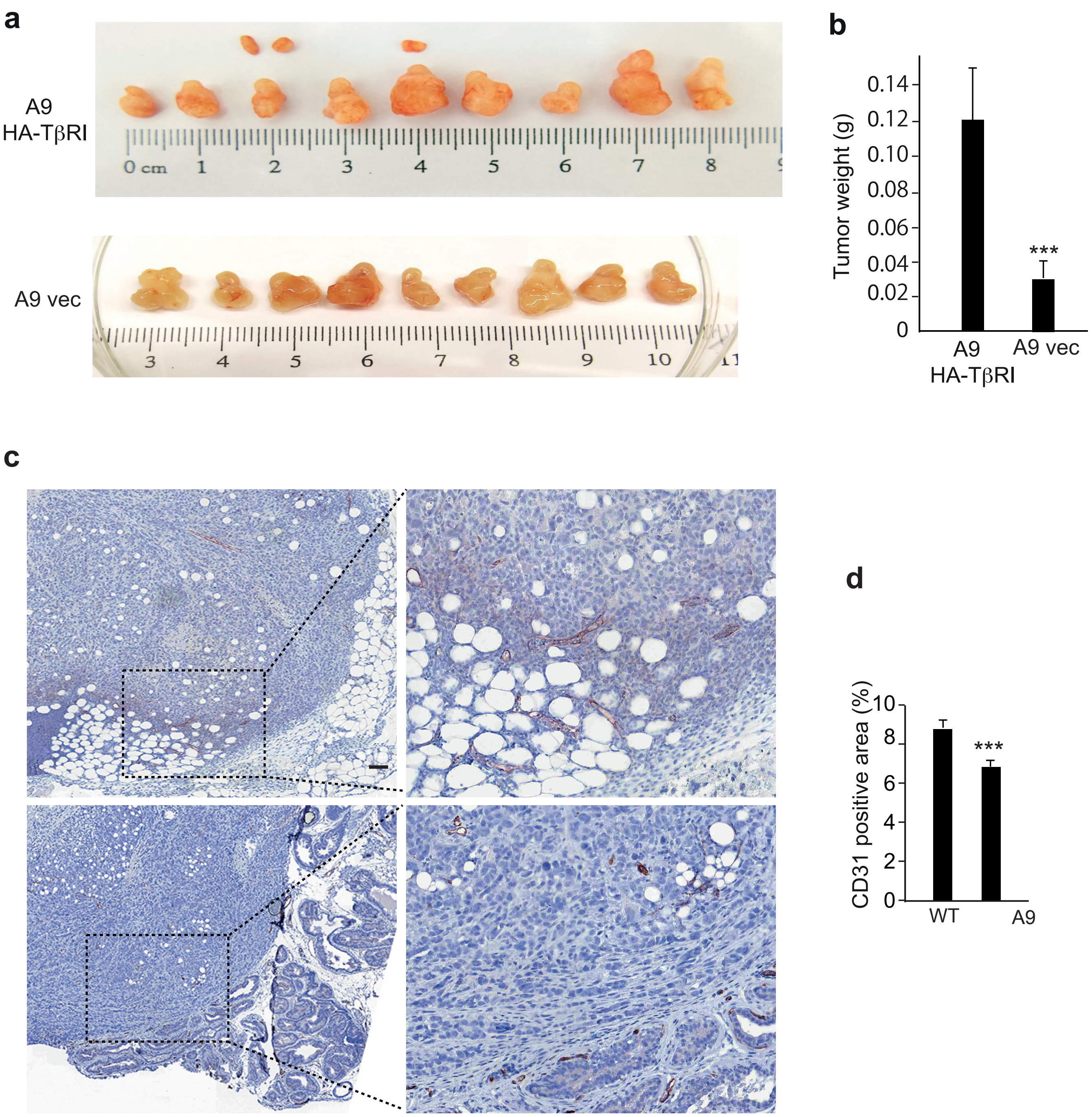

Fig. S3. (a, b) A9 cells reconstituted with HA-TβR1 partially rescued the non-invasive phenotype in vivo. A9 with HA-TβR1 group: tumor, n=9; LN, n=3. A9 with vectors: tumor, n=9; LN, n=0. Results are shown as mean ± SEM, *** $p < 0.001$, Student's t test. (c) CD31 staining of WT and A9 tumors. (d) Vascular density was evaluated as CD31 positive area in WT (n=7) and A9 (n=6) tumors. Results are shown as mean ± SEM, *** $p < 0.001$, Student's t test.
